# Clinical characteristics and prognosis of acute low-frequency hearing loss and ascending sensorineural sudden sensorineural hearing loss

**DOI:** 10.3389/fnins.2022.1076109

**Published:** 2023-01-10

**Authors:** Tongxiang Diao, Yurun Chen, Yuanyuan Jing, Xin Ma

**Affiliations:** Department of Otolaryngology, Head and Neck Surgery, People’s Hospital, Peking University, Beijing, China

**Keywords:** ALFHL, ascending SSNHL, audiogram shape, prognosis, related factors

## Abstract

**Objective:**

The present study aimed to explore the pathogenesis of the ascending sudden sensorineural hearing (SSNHL) loss by comparing the clinical characteristics and prognosis of acute low-frequency hearing loss (ALFHL) and ascending SSNHL.

**Methods:**

A total of 43 patients with ALFHL and 122 patients with ascending SSNHL were enrolled in this study. First, the prognosis of patients with ALFHL and ascending SSNHL were compared, and the prognostic factors of AFHL and ascending SSNHL were analyzed.

**Results:**

Acute low-frequency hearing loss and ascending SSNHL have no remarkable difference in complete recovery rate. Compared to ascending SSNHL, ALFHL has younger onset age, female prevalence, lower hearing threshold, shorter time from onset to recovery, and a lower proportion of combined tinnitus. The PTA at admission and delay from onset to therapy were significantly related to the prognosis of patients with ascending SSNHL, while only delay from onset to therapy was significantly related to the prognosis of patients with ALFHL. The majority of patients with ascending SSNHL and ALFHL recovered completely within 10 days from onset.

**Conclusion:**

Audiogram shape plays a critical role in the prognosis of SSNHL. Ascending SSNHL and ALFHL may share a common pathological mechanism.

## Introduction

Acute low-frequency sensorineural hearing loss (ALFHL) has been interpreted as an independent disease entity distinct from idiopathic sudden sensory neural hearing loss (SSNHL) (Imamura et al., 1997), characterized by low-frequency hearing loss, better prognosis, and high recurrence rate (Fuse et al., 2002). The diagnostic criteria for ALFHL were as follows: (1) acute onset sensorineural hearing loss of >30 dB at two consecutive low frequencies (250 and 500 Hz) within a period of 3 days; (2) 25 dB of normal hearing at hearing thresholds of 1, 2, 3, 4, and 8 kHz on the affected side; (3) 25 dB of normal hearing at hearing thresholds of 250, 500, 1,000, 2,000, 4,000, and 8,000 Hz on the unaffected side; and (4) no history of episodic dizziness or spontaneous nystagmus (Jung et al., 2016). The pathophysiological mechanisms of ALHL are similar to those of sudden hearing loss; this phenotype has been associated with cochlear hydrops (CH) and early stages of Meniere’s disease (Yamasoba et al., 1993). Due to the presence of helicotrema, hydrops begin at the apical turn of the cochlea, manifesting as low-frequency deafness, subsequently involving all frequencies with worsening hydrops (Thai-Van et al., 2001).

Sudden sensory neural hearing loss is defined as at least 30 dB sensorineural hearing loss in three sequential frequencies within 3 days with no identifiable cause (Stachler et al., 2012), which has an incidence ranging from 5 to 27/100,000 people in the Western countries and 19/100,000 in mainland China (Xie et al., 2020). The pathogenesis of SSNHL is yet unknown, although several hypotheses have been proposed, including viral infection, vascular compromise, membrane labyrinth hydrops, chronic inflammation, immunological diseases (Rossini et al., 2017), cochlear membrane rupture (Kuhn et al., 2011), inner ear cell stress reaction (Merchant et al., 2005), hemorrhage of the inner ear (Berrettini et al., 2013), and migraine (Hwang et al., 2018). Some studies have proposed that the SSNHL can be divided into four types of audiogram shapes based on the pattern of hearing loss: ascending, flat, profound, and descending. “Ascending or descending” referred to cases in which the average threshold of 0.25–0.5 kHz was 20 dB higher or lower than the average threshold of 4–8 kHz. When the difference in hearing threshold did not exceed 20 dB at any frequency, the audiogram shape was classified as “flat.” For patients with a flat audiogram and hearing threshold >90 dB, the audiogram shape was classified as “profound.” As shown previously, the ascending type has the best prognosis (Mattox and Simmons, 1977; Qian et al., 2018), with a recovery rate of about 83.33% (Li et al., 2016). The present study aimed to explore the pathogenesis of the ascending SSNHL by comparing the clinical characteristics and prognosis of patients with ALFHL.

## Materials and methods

### Study design and inclusion and exclusion criteria

This retrospective cohort study included 43 patients in line with the definition of ALFHL and 122 patients with ascending SSNHL hospitalized in our department for treatment from July 2015 to May 2018.

#### Inclusion criteria

Ascending SSNHL group: (1) 18–70-year-old; (2) no gender requirement; (3) first-onset SSNHL; (4) unilateral hearing loss; (5) time from onset to treatment ≤14 days; (6) the average threshold of 0.25–0.5 kHz was 20 dB higher than the average threshold of 4–8 kHz, and all frequency hearing thresholds were >25 dB; and (7) normal hearing or age-related hearing loss in the contralateral ear.

Acute low-frequency hearing loss group: (1) 18–70-year-old; (2) no gender requirement; (3) first-onset SSNHL; (4) unilateral hearing loss; (5) a time between onset and treatment ≤14 days; (6) hearing loss of >30 dB at two consecutive low frequencies (250 and 500 Hz), but with 25 dB of normal hearing at hearing thresholds of 1, 2, 3, 4, and 8 kHz on the affected side; and (7) normal hearing or age-related hearing loss in the contralateral ear.

#### Exclusion criteria

(1) Pregnancy or middle ear infections; (2) a definitive cause of deafness identified during treatment; (3) acoustic neuroma and other organic diseases; and (4) anxiety and insomnia.

### Treatment

After admission, treatments were applied in accordance with the Editorial Board of Chinese Journal of Otorhinolaryngology Head and Neck Surgery; Society of Otorhinolaryngology Head and Neck Surgery, Chinese Medical Association (2015). The systemic corticosteroids and hemodilution agents were administered as therapeutic measures. All patients received 40 mg of intravenous methylprednisolone for 5 consecutive days and hemodilution agents for 10 days, including 87.5 mg of intravenous EGb-761 (Ginkgo Biloba Extracts) (Dr. Willmar Schwabe GmbH & Co., Germany) daily.

### Efficacy evaluation

Pure-tone audiometry was performed at the initial presentation and 1 month after treatment. The pure-tone average of all frequencies (250, 500, 1,000, 2,000, 4,000, and 8,000 Hz) was employed to determine the treatment outcomes according to Siegel’s (1975) criteria. Complete recovery (CR) indicated that the “final hearing level was <25 dB.” Final hearing at 25–45 dB with a hearing gain of ≥15 dB was “partial recovery.” “Slight recovery” meant final hearing >45 dB with hearing gain of ≥15 dB. The final hearing level >75 dB with hearing gain of ≤15 dB represented “no recovery.”

### Statement of ethics

This cohort research was approved by the Peking University People’s Hospital Ethical permission committee (2021PHB149). Written informed consent for this research was received from each patient.

### Statistic

The clinical and epidemiological characteristics of the patients were summarized by descriptive statistics. The datasets were described with median and/or range. Numerical data were compared using *t*-test, and categorical data were compared using the χ^2^ test. First, univariate analysis was used to compare the epidemiological and clinical characteristics of ALFHL and ascending SSNHL group, and then binary logistic regression analysis was used to analyze the prognostic factors of ALFHL and ascending SSNHL. The risk ratios were presented with 95% confidence interval (CI). Statistical significance was defined as a two-tailed *P* < 0.05 for all analyses. Statistical analyses were performed using SPSS software version 23.0 and GraphPad Prism 7.0.

## Results

### Clinical characteristics of ascending SSNHL and ALFHL

A total of 43 ALFHL patients and 122 patients with ascending SSNHL were enrolled in this study. Independent sample *t*-test and *c*^2^-square test were used to compare the clinical characteristics and CR rate between patient groups with ascending SSNHL and ALFHL (Table 1). The statistical results showed statistical differences in age, gender, PTA at admission (the average hearing thresholds of 0.25, 0.5, 1.0, 2.0, 4.0, and 8.0 kHz), 1 kHz pure-tone threshold, 2 kHz pure-tone threshold, tinnitus occurrence, and the delay from onset to CR (onset-CR) between the two groups; however, the CR was not statistically different. Compared to the ascending SSNHL group, patients with ALFHL had a lower age of onset, lower PTA at admission, lower pure-tone threshold at 1 and 2 kHz, female tendency, shorter onset-CR time, and lower tinnitus incidence.

**TABLE 1:** Clinical characteristics of ascending sudden sensorineural hearing loss (SSNHL) and acute low-frequency hearing loss (ALFHL).

	Ascending SSNHL (*n* = 122)	ALFHL (*n* = 43)	*p*
Age	38.35 ± 12.89	33.33 ± 10.07	0.011*
Sex (female)	62/122 (50.82%)	31/43 (72.09%)	0.020*
Lateral (left)	64/122 (52.46%)	21/43 (48.84%)	0.725
PTA (dB)	44.48 ± 19.07	20.23 ± 4.25	0.000*
Tinnitus	111/122 (90.98%)	33/43 (76.74%)	0.020*
Dizziness	18/122 (14.74%)	12/43 (27.91%)	0.067
Aural fullness	63/122 (51.64%)	25/43 (58.14%)	0.483
Headaches	8/122 (6.56%)	1/43 (2.33%)	0.448
CR rate	84/122 (68.85%)	34/43 (79.07%)	0.241
UR rate	12/122 (9.84%)	4/43 (9.30%)	1.000
Onset-CR			0.004*
3 days	22/122 (18.03%)	20/43 (46.51%)	
3–7 days	38/122 (31.15%)	7/43 (16.28%)	
6–10 days	19/122 (15.57%)	5/43 (11.63%)	
10–14 days	5/122 (4.10%)	1/43 (2.33%)	
14–30 days	0/122 (0.00%)	1/43 (2.33%)	
Un-CR	38/122 (31.15%)	9/43 (20.93%)	
Onset-therapy	3.41 ± 3.12	3.47 ± 2.30	0.920

CR, complete recovery; UR, un-recovery.

*p < 0.05.

### Factors related to the prognosis of ALFHL

A total of 34/43 patients with ALFHL (79.07%) recovered completely. First, the univariate analysis showed that delay from onset to therapy (onset-therapy) was statistically related to the prognosis of patients with ALFHL. Then, onset-therapy and clinically significant factors, including age, PTA at admission, tinnitus, and dizziness, were included in the binary logistic regression analysis. The results showed that delay from onset-therapy was an independent related factor to the prognosis of ALFHL (Table 2).

**TABLE 2:** Factors related to prognosis of acute low-frequency hearing loss (ALFHL).

Variable	Un-CR (*n* = 9)	CR (*n* = 34)	*p*
**Univariate analysis**			
Age	37.67 ± 10.559	32.18 ± 9.778	0.145
Onset-therapy	6.44 ± 4.613	2.68 ± 1.788	0.041*
PTA at admission	21.389 ± 4.167	19.927 ± 4.286	0.365
Gender (female)	6/9 (66.67%)	25/34 (73.53%)	0.692
Lateral (left)	5/9 (55.56%)	16/34 (47.06%)	0.721
Tinnitus	8/9 (88.89%)	25/34 (73.53%)	0.659
Dizziness	5/9 (55.56%)	7/34 (20.59%)	0.088
Aural fullness	6/9 (66.67%)	19/34 (55.88%)	0.712
Headache	1/9 (11.11%)	0/34 (0.00%)	0.209
**Multivariate analysis**			
	OR (95%CI)		*p*
Onset-therapy (days)	0.651 (0.463–0.915)		0.013*

*p < 0.05.

### Factors related to the prognosis of ascending SSNHL

A total of 118/122 patients with ascending SSNHL (68.85%) completely recovered. First, the univariate analysis showed that delay from onset-therapy was statistically related to the prognosis of patients with ascending SSNHL. Then, onset-therapy and clinically significant factors, including age, PTA at admission, tinnitus, and dizziness, were included in the further binary logistic regression analysis. The results showed that PTA at admission and delay from onset-therapy were independent factors of the prognosis of ascending SSNHL (Table 3).

**TABLE 3:** Factors related to prognosis of ascending sudden sensorineural hearing loss (SSNHL).

Variable	Un-CR (*n* = 38)	CR (*n* = 84)	*p*
**Univariate analysis**			
Age	40.08 ± 14.143	37.57 ± 12.283	0.322
Onset-therapy	4.82 ± 4.591	2.77 ± 1.865	0.011*
PTA at admission	46.84 ± 19.723	43.414 ± 18.785	0.360
Gender (female)	19/38 (50%)	43/84 (51.19%)	0.903
Lateral (left)	20/38 (52.63%)	44/84 (52.38%)	0.980
Tinnitus	36/38 (94.74%)	75/84 (89.29%)	0.330
Dizziness	7/38 (18.42%)	11/84 (13.10%)	0.442
Aural fullness	17/38	46/84	0.333
Headache	1/38	7/84	0.433
**Multivariate analysis**			
	OR (95%CI)		*p*
PTA at admission	0.981 (0.960–1.003)		0.092
Onset-therapy (days)	0.781 (0.671–0.909)		0.001*

*p < 0.05.

### Distribution of onset-CR of ALFHL and ascending SSNHL

**FIGURE 1:**
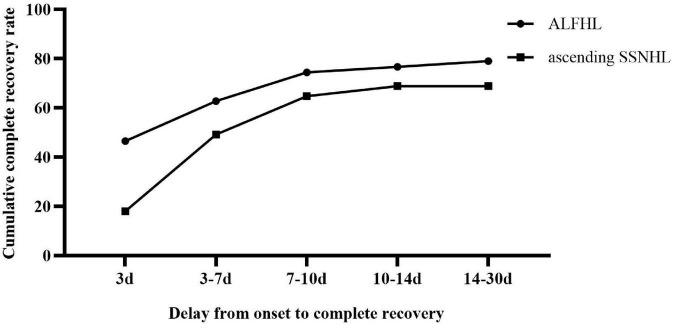
Distribution of onset-CR of acute low-frequency hearing loss (ALFHL) and ascending sudden sensorineural hearing loss (SSNHL).

The CR of ALFHL was 79.07% (34/43), of which 94.12% (32/34) were recovered within 10 days after onset. The CR of ascending SSNHL was 68.85% (84/122), which was slightly lower than that of ALFHL, albeit not significantly. Among them, 64.75% recovered within 10 days after the onset, accounting for 94.05% (79/84) of all recovered patients (Figure 1). Therefore, both ALFHL and ascending SSNHL were recovered within 10 days after onset, suggesting that both ALFHL and ascending SSNHL might lie in common pathogenesis. It also suggested that one must have reasonable expectations of patients who have not completely recovered after 10 days from onset (Table 4).

**TABLE 4:** Distribution of onset-CR of patients with ascending sudden sensorineural hearing loss (SSNHL) and acute low-frequency hearing loss (ALFHL).

		3 days	3–7 days	7–10 days	10–14 days	14–30 days
ALFHL	Cases	20/43	7/43	5/43	1/43	1/43
	CR rate	46.51%	16.28%	11.63%	2.33%	2.33%
	Cumulative frequency	46.51%	62.79%	74.42%	76.74%	79.07%
Ascending SSNHL	Cases	22/122	38/122	19/122	5/122	0/122
	CR rate	18.03%	31.15%	15.57%	4.10%	0.00%
	Cumulative frequency	18.03%	49.18%	64.75%	68.85%	68.85%

## Discussion

The present study found that compared to ascending SSNHL, patients with ALFHL had a lower age of onset, lower PTA at admission, lower pure tone threshold at 1 and 2 kHz, female tendency, shorter time from onset to recovery, and a tinnitus incidence; however, the CR was not statistically different.

### No difference in the CR between ascending SSNHL and ALFHL

Several studies showed that audiogram shape is a major prognostic factor of SSNHL, divided into four clinical types: ascending, descending, flat, and profound (Chung et al., 2015). These factors guide the prognosis of patients with SSNHL.

Among all the four types, the ascending type has the best prognosis: 63–88% (Watanabe and Suzuki, 2018). Similarly, patients with ALFHL also showed a good prognosis. Typically, ALFHL has a better prognosis than SSNHL and responds well to treatment. Some studies reported that 32–65% of ALFHL patients recover their hearing spontaneously without treatment (Mattox and Simmons, 1977; Conlin and Parnes, 2007). Choo et al. (2017) found that about 70% of ALFHL patients treated with steroid therapy had recovered hearing. Nonetheless, whether ALFHL is an independent disease is yet controversial. Some studies considered ALFHL as a subtype of SSNHL, which has a better prognosis than other types (Shaia and Sheehy, 1976; Mattox and Simmons, 1977). In contrast, because of the good prognosis, high recurrence rate, ease of development into MD, and some other clinical characteristics, some studies classified it as a type of disease independent of SSNHL.

The results of this study did not present any significant difference in the CR between patients with ALFHL and ascending SSNHL. Compared to ascending SSNHL, patients with ALFHL were younger and with less severe hearing loss. In addition, ALFHL might be caused by various causes, among which CH is widely recognized (Noguchi et al., 2004; Junicho et al., 2008). It is illustrated that the apical turn of the cochlea is more sensitive to pressure changes than the basal turn; hence, the inner ear hydrops begins at the apical turn of the cochlea and subsequently extends to the cochlear aqueduct and the vestibular apparatus (7). Thus, ALFHL may be the early stage of ascending SSNHL; with aggravated inner ear hydrops, the degree of hearing loss is also aggravated and combined symptoms, resulting in tinnitus. The pathogenesis of ascending SSNHL is similar to ALFHL and might be related to CH (Junicho et al., 2008; Editorial Board of Chinese Journal of Otorhinolaryngology Head and Neck Surgery; Society of Otorhinolaryngology Head and Neck Surgery, Chinese Medical Association, 2015).

Furthermore, in view of the similar prognosis between ascending SSNHL and ALFHL, for patients with ascending SSNHL, partial treatment can be reduced, which is of great significance for optimizing medical resources. Because the etiology is usually unknown, treatments have been empiric (Wei et al., 2013). The lack of one or more uniformly accepted treatment(s) potentially decreases the cost of management.

### Factors related to the prognosis of ALFHL and ascending SSNHL

Factors affecting prognosis in patients with SSNHL include delay from onset-therapy, the occurrence of dizziness or tinnitus, type of audiogram, and initial hearing loss (Bespalova et al., 2001). Consistent with these findings, the current study showed that PTA at admission and delay from onset-therapy were independent factors related to the prognosis of ascending SSNHL. Many studies speculated that the earlier the patient receives treatment, the better the prognosis (Shaia and Sheehy, 1976; Byl, 1984). Byl (1984) demonstrated that when patients receive treatment within 7 days from onset, the recovery rate is 56%, while the recovery rate of patients who received treatment for >30 days after onset is only 27%. Shaia and Sheehy (1976) also proposed that the prognosis is improved when patients are treated within 30 days from onset. Moreover, the severity of hearing loss was a major prognostic factor. In the present study, the PTA at admission is an independent prognostic factor for patients with ascending SSNHL. Conversely, in patients with ALFHL, PTA at admission is not related to prognosis, which might be due to mild hearing loss. In the case of delay from onset to complete recovery, Sano et al. (1998) speculated that patients with hearing loss <70 dB at admission are likely to heal within 8 days, which is consistent with the results of this study, i.e., most patients with ascending sudden SSNHL and ALFHL recovered within 10 days (70.3%, 116/165).

### Limitations

Nevertheless, the present study also has some limitations. First, this retrospective analysis caused information bias in the statistical analysis. Second, this study collected patients with ascending SSNHL and ALFHL admitted to the hospital; however, several patients with ascending SSNHL, especially with ALFHL, would prefer to receive outpatient treatment because of mild hearing loss, causing a certain degree of selection bias. Third, the follow-up of this study was short, i.e., followed up to 1 month after treatment, making it impossible to collect data on the long-term prognosis and recurrence of patients with ascending SSNHL and ALFHL; these would be assessed in our follow-up studies.

## Conclusion

The PTA at admission and delay from onset-therapy were independent factors related to the prognosis of ascending SSNHL, while only the delay from onset-therapy was remarkably related to the prognosis of ALFHL. Audiogram shape is a prognostic factor of SSNHL; also, no statistical difference was observed in the CR rate between ALFHL and ascending SSNHL, which manifests as a similar audiogram shape, suggesting that ascending SSNHL and ALFHL share some common pathological mechanisms.

## Data availability statement

The raw data supporting the conclusions of this article will be made available by the authors, without undue reservation.

## Ethics statement

The studies involving human participants were reviewed and approved by the Peking University People’s Hospital Ethical Permission Committee. The patients/participants provided their written informed consent to participate in this study.

## Author contributions

XM designed and conceptualized this study, interpreted the data, and revised the manuscript. TD, YC, and YJ designed and conceptualized study, collected and analyzed the data, and drafted the manuscript for intellectual content. All authors contributed to the article and approved the submitted version.
